# Assessing the environmental destruction in forest ecosystems using landscape metrics and spatial analysis

**DOI:** 10.1038/s41598-023-42251-6

**Published:** 2023-09-13

**Authors:** M. Mansori, Z. Badehian, M. Ghobadi, R. Maleknia

**Affiliations:** 1 Faculty of Natural Resources, Lorestan University, Khorramabad, Iran; 2 Faculty of Agriculture, Fasa University, Fasa, Iran

**Keywords:** Ecology, Environmental sciences

## Abstract

Forest degradation is a serious environmental issue that has significant implications for ecological stability, biodiversity, and human well-being. Identifying the extent and severity of forest degradation is crucial for effective management and conservation of forest ecosystems. The objective of this study was to assess the ecological vulnerability of the forest in the Dadabad region using spatial analysis and landscape metrics. The land cover map of the area was divided into 13 sub-basins to quantify landscape metrics, and the severity of human activities, degradation level, and ecological vulnerability were calculated for each sub-basin. Each sub-basin was evaluated as a single landscape to determine the extent of degradation, and landscape zoning was performed based on the degradation coefficients. The study found that sub-basins 2 and 4 had the highest levels of degradation, while sub-basins 3, 7, 8, 11, and 12 were the least degraded. Over half of the Dadabad region, 37.8% for protection and 32.25% for rehabilitation, was recommended for conservation planning. The research highlights the importance of using spatial analysis of landscape metrics to assist managers and planners in protecting and conserving natural areas.

## Introduction

Forests are crucial ecosystems that support a wide range of ecological functions and provide numerous benefits to human societies, including timber, non-timber forest products, and ecosystem services such as carbon sequestration and regulation of water resources^1^. However, forests around the world are facing increasing threats from deforestation, degradation, and fragmentation, which have significant impacts on biodiversity, ecosystem services, and human livelihoods^2^. Land use changes and degradation of forests are significant environmental issues that have attracted increasing attention in recent decades. These issues are primarily driven by human activities such as urbanization, industrialization, and agriculture expansion. The consequences of these activities include fragmentation and simplification of natural ecosystems, soil erosion, loss of biodiversity, and changes in hydrological regimes, among others^3^. The severity of these problems varies globally, and the impacts are often long-lasting, affecting both the environment and human well-being^4^.

Forests, in particular, are among the most important natural resources on earth, providing habitat for biodiversity, regulating water and carbon cycles, and supporting human livelihoods^2,4^. Forest degradation and deforestation have been identified as significant contributors to climate change, accounting for approximately 10% of global greenhouse gas emissions^1,5^. Forest degradation can occur due to various factors, such as logging, fire, and invasive species, among others^6–8^. These factors often lead to a loss of forest structure and function, which can reduce forest productivity and ecological services^7^. Land use changes, on the other hand, result from the conversion of natural ecosystems into human-dominated landscapes^8^. Such changes can alter the ecological processes that support biodiversity and ecological services, leading to degradation and fragmentation of natural habitats^9^. Land use changes often occur in developing countries, where there is a high demand for land for agriculture, urbanization, and infrastructure development. In developed countries, land use changes often result from urban sprawl, which leads to fragmentation and loss of natural habitats. These issues have been recognized globally, and efforts have been made to address them through various policies and initiatives. For instance, the United Nations Framework Convention on Climate Change (UNFCCC) has developed mechanisms such as Reducing Emissions from Deforestation and Forest Degradation (REDD+) to reduce greenhouse gas emissions from forest degradation and deforestation^3^. In addition, various sustainable land-use practices, such as agroforestry and sustainable forestry, have been implemented to address the challenges of land use changes and forest degradation. Despite these efforts, the challenges of land use changes and forest degradation continue to persist. Therefore, it is essential to understand the drivers and impacts of these issues and develop appropriate strategies to address them. This can be achieved through the use of spatial analysis and landscape metrics, which provide a quantitative tool for assessing the ecological vulnerability of natural areas and forests. Such tools can help identify areas that require protection, rehabilitation, or sustainable land-use practices^10^. One approach for assessing forest degradation is through the use of landscape metrics, which are quantitative measures of the spatial patterns of land cover and land use. Landscape metrics can provide valuable insights into changes in forest structure and composition, fragmentation, and connectivity, which are important indicators of forest degradation^9–12^. By analyzing landscape metrics, researchers and conservation practitioners can identify areas of degraded forest and develop appropriate management strategies to restore and conserve forest ecosystems^11^. Landscape metrics provide a powerful tool for analyzing the spatial patterns of land cover and land use, and for identifying areas of degraded forest^13–15^. By quantifying landscape structure, fragmentation, and connectivity, landscape metrics can reveal patterns of forest degradation and guide the development of appropriate management strategies^16^. There are numerous landscape metrics that can be used to assess forest degradation, including measures of patch size, shape, and connectivity, as well as measures of edge density and landscape diversity^17,18^. These metrics can be calculated using remotely sensed data, such as satellite imagery, and can be used to identify areas of degraded forest and prioritize restoration efforts^19^. This paper aims to evaluate the ecological vulnerability of the forest landscape in the Dadabad area using spatial analysis and landscape metrics. The findings of this study can assist managers and planners in developing appropriate conservation and land-use plans in natural areas.

## Materials and methods

### Study area

The study was conducted in the Dadabad forest area located in the Lorestan province of western Iran, spanning from longitude 24° 32^/^ 60^//^ to 23° 16^/^ 64^//^ E and latitude 37° 68^/^ 90^//^ to 36° 83^/^ 30^//^ N. The area experiences a mean annual rainfall of approximately 561.43 mm, with the maximum 24-h rainfall reaching 77.84 mm. The climate in this region is influenced by both altitude and Mediterranean conditions, with a semi-arid climate prevailing. The altitude of the region is around 1450 m, leading to significant variations in temperature and land cover.

**Figure 1:**
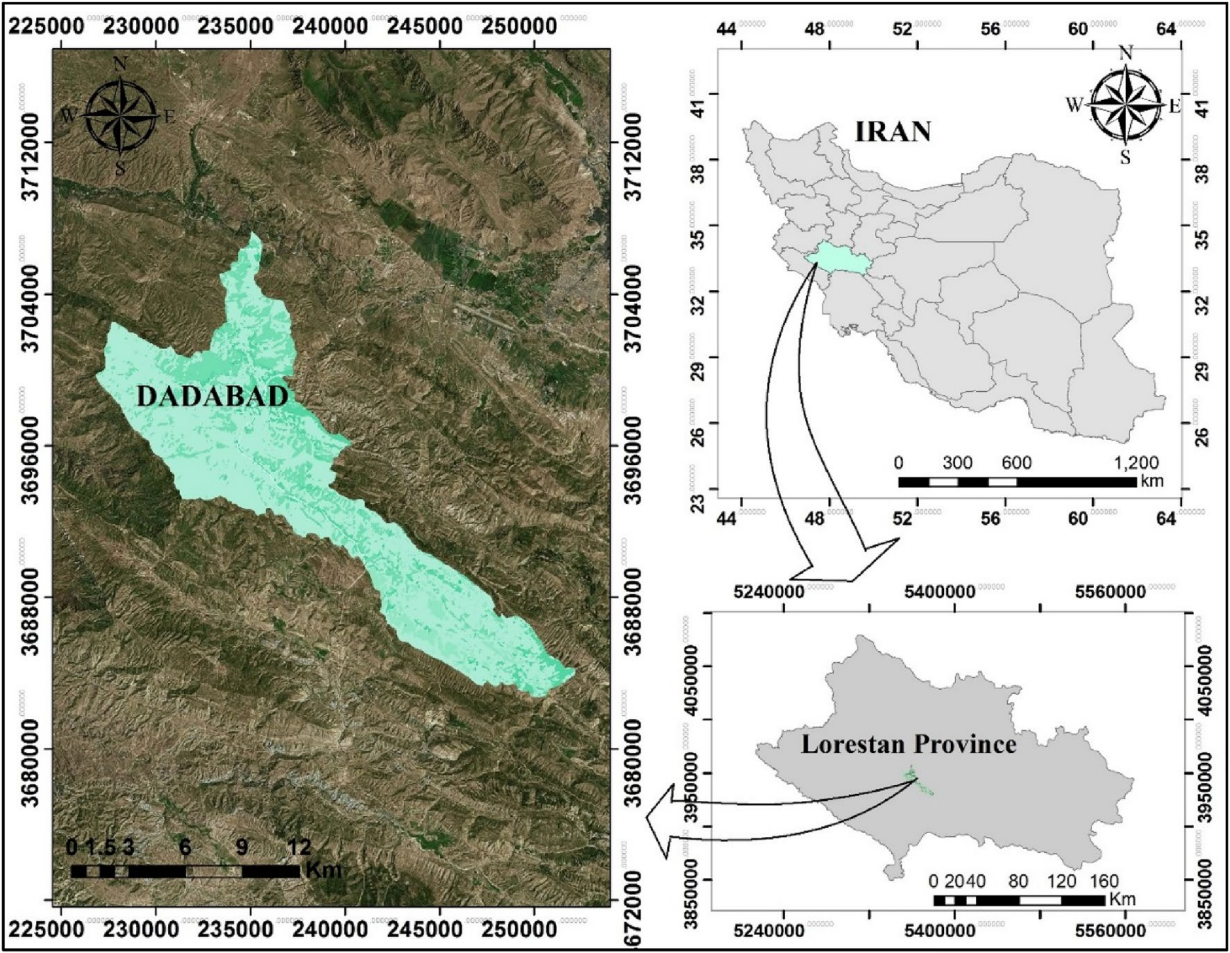
Location of the study area.

The geographic location of the study area is showed in Fig. 1, generated using GIS software version 10.8 and the ArcBruTile 0.7 extension to display the background image from Google Earth’s satellite imagery.

### Data preparation

To evaluate the changes in the study area’s structure, a land cover map was derived from satellite images. In order to obtain accurate results, data from the growing seasons was utilized. The landscape satellite image had a spatial resolution of 30 m and was acquired in 2022. A maximum likelihood algorithm was employed to classify the landscape patterns into six categories, namely forest, agriculture, barren lands, grasslands, villages, and roads, using TerrSet software. To assess the classification accuracy, 300 random training points were extracted from land use maps and Google Earth images. The dataset was chosen in a way that they represent a diverse range of locations within the study area. These points were manually identified and labeled by human experts who visually inspected the land use maps and Google Earth images. Each point was assigned a specific class label based on the predominant land cover type in its vicinity. This process ensured that the ground truth dataset accurately reflected the distribution and characteristics of the different land cover classes. The accuracy of the satellite image in the study was evaluated using the Kappa coefficient and overall accuracy metrics. These metrics were utilized to determine the level of agreement between observed and expected classifications, as well as to measure the proportion of correctly classified pixels or areas. By applying these metrics, the study aimed to provide a thorough and comprehensive assessment of the accuracy of the satellite image. The image was obtained from Landsat 8 Satellite and OLI sensor, with row and path numbers of 166/37, and a resolution of 15 m panchromatic and 30 m of spatial resolution. The sub-domain map was used as input in FRAGSTATS software, and the results were stored and analyzed.

All maps were meticulously generated using the powerful capabilities of the geographic information system (GIS) software version 10.8. GIS software is a sophisticated tool that allows us to capture, analyze, interpret, and visualize spatial data, enabling us to represent geographical information in a comprehensive and visually engaging manner.

### Calculating the destruction rate

The destruction rate refers to the speed or rate at which forests are being destroyed or lost^6^. It is typically expressed as the percentage of forest area lost over a specific time period^17,20^. The destruction rate is a crucial measure to evaluate the scale and impact of human activities and natural events on forest ecosystems^7^. Monitoring and measuring the destruction rate in forests is essential for assessing the effectiveness of conservation policies and interventions^8^. It helps policymakers and researchers understand the causes and consequences of deforestation, identify areas at high risk of forest loss, and develop strategies to mitigate and prevent further degradation^13^. To estimate the destruction rate, satellite imagery, remote sensing technologies, and on-the-ground surveys are commonly used. These methods provide valuable data on changes in forest cover and land use, allowing scientists to calculate the rate of forest loss and identify regions or specific activities contributing to destruction^20^. Equation (1)^13,20^ incorporates all variables that affect degradation into the destruction equation.

1 $$ {\text{Hi}} = \, \left( {\sum {\text{I}} + {\text{DPi}}} \right)/{\text{Vi}} $$

The degradation coefficient in selected units, Hi, is determined by the collective effects of destruction factors in the past (ΣI), the physiological density in units up to i (DPi), and the degree of vulnerability of the habitat in unit i (V).

2 $$ {\text{LD}} = \sum {\text{ki}}/{\text{Vi}} $$

In Eq. (2)^20^, the destruction coefficient (LD) is calculated by summing the index of the intensity of human activities in the destruction of the landscape (Σki) divided by the degree of vulnerability of the habitat in unit i (Vi). The proposed model categorizes destruction classes based on the region’s ecological values, with the natural ecosystem classified as a vulnerable class. The intensity of human activity, degree of ecological vulnerability, and limit of the landscape destruction classes were determined based on the median of the data.

### Landscape metrics for assessing the destruction

In the selection of landscape structural metrics for the destruction model, several considerations were taken into account. Relevance to the research objectives was prioritized to capture the landscape’s key characteristics affecting destruction. A comprehensive review of existing literature identified widely used and informative metrics. Metrics calculable with commonly available spatial data were preferred for their feasibility. The interpretability and ecological significance of metrics were evaluated to select those providing insights into the underlying processes. These factors ensure the chosen metrics are appropriate and relevant, shedding light on the landscape characteristics impacting destruction and enhancing understanding of the phenomenon. Landscape fragmentation, which divides land into smaller pieces, is one of the crucial processes affecting landscape structure and function^21^. Landscape metrics can be used to quantitatively measure landscape processes, and they are valuable for incorporating ecosystem perspectives into environmental programs^21,22^. Table 1 describes the selected metrics as variables of landscape destruction.

**Table 1:** Landscape structural metrics in the destruction model.

Metric	Description	Unit	Range
NP	Number of patches	–	NP ≥ 1
MPS	Mean patch size	ha	MPS ≥ 0
MedPS	Middle patch Size	ha	MedPS ≥ 0
TE	Total edge	m	TE ≥ 0
ED	Edge density	m/ha	ED ≥ 0
FDI	Fractal dimension index	–	1 ≦ FDI ≦ 2
SDI	Shannon’s diversity	–	SDI ≥ 0
MSI	Mean shape index	–	MSI ≥ 0

**Figure 2:**
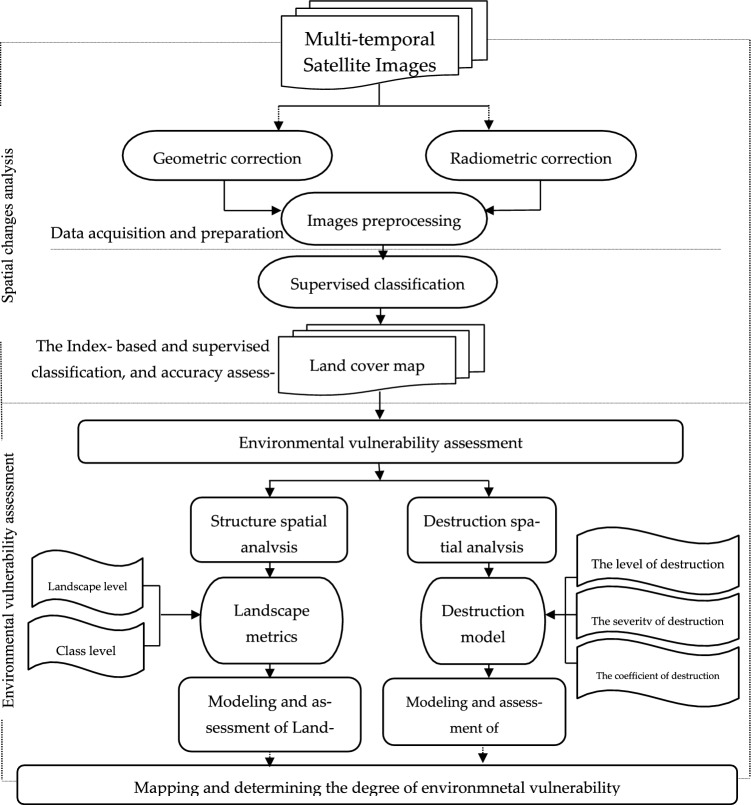
Flowchart of the research.

Initially, the study identified metrics for sub-basins, followed by determining the range of each metric based on sub-basins and data median. Then, the study added the destruction code of these metrics and considered them as a set of destructive factors in the landscape. The severity of destruction was determined qualitatively by categorizing the codes into minor damage, moderate damage, severe damage, and very severe damage^22,23^. Finally, the study compared the metric coefficients in each Landscape unit with the median scale to determine the intensity of each activity among work units. Figure 2 illustrates the flowchart of the research.

## Results

**Figure 3:**
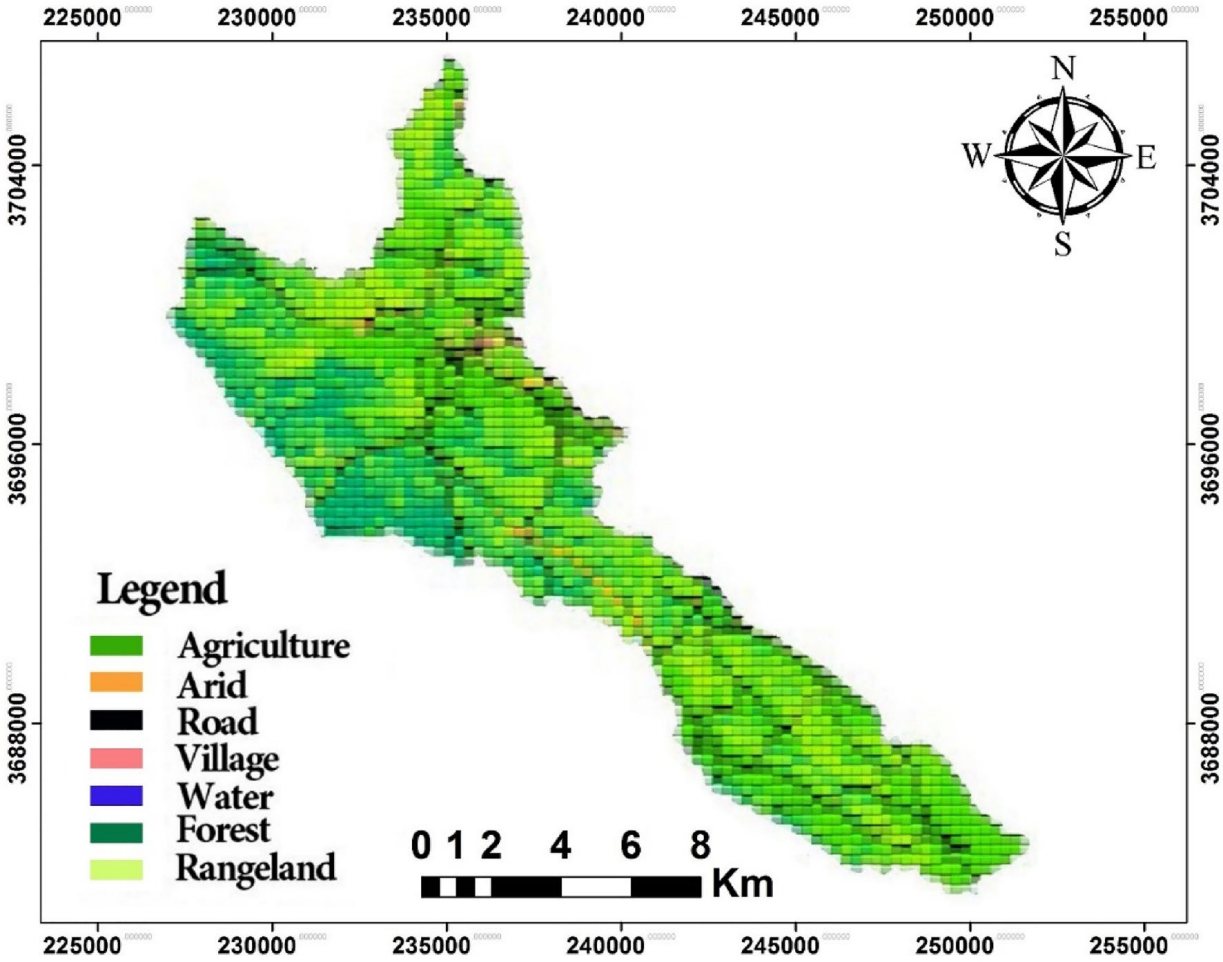
Land cover map of the Dadabad area.

The map that was created consists of six categories: forest, agriculture, arid land, rangeland, village, and road. These categories were utilized to determine Landscape metrics. The evaluation of the classification demonstrated that the overall accuracy and Kappa coefficient were 90.4 and 0.88 respectively. This indicates a high level of reliability. Figure 3 illustrates the classification map of the Dadabad forest region.

Table 2 displays the results of calculating metrics for the 13 sub-subdivisions of the region, with each cell being treated as a subsystem, in order to assess the extent of damage to the entire landscape.

**Table 2:** Categories landscape metrics in each sub-system.

Metric	NP		MPS		MedPS		ED		TE		MFPD		MSI		SDI	
	1149 ≥ X_4_		2/25 ≥ X_1_		0/25 ≥ X_1_		300 ≥ X_4_		767 ≥ X_4_		1/14 ≥ X_4_		1/17 ≥ X_4_		1/20 ≥ X_4_	
	> 702 ≥ ›X_3_		> 1/89 ≥ ›X_2_		> 0/79 ≥ ›X_2_		> 175 ≥ ›X_3_		> 423 ≥ ›X_3_		> 1/09 ≥ › X_3_		> 1/09 ≥ X_3_		> 1/08 ≥ ›X_3_	
	> 405 ≥ ›X_2_		> 1/04 ≥ ›X_3_		> 1/58 ≥ ›X_3_		> 105 ≥ ›X_2_		> 241 ≥ ›X_2_		> 1/04 ≥ ›X_2_		> 1/05 ≥ ›X_2_		> 1/02 ≥ ›X_2_	
	> X_1_		> X_4_		> X_4_		> X_1_		> X_1_		> X_1_		> X_1_		> X_1_	
Sub-basin	Quantity	Intensity	Quantity	Intensity	Quantity	Intensity	Quantity	Intensity	Quantity	Intensity	Quantity	Intensity	Quantity	Intensity	Quantity	Intensity
1	900	I3	2.21	I2	2	I4	227	I3	211	I1	1.09	I3	1.2	I1	1.03	I2
2	402	I1	0.38	I4	0.35	I1	158	I2	240	I1	1.04	I2	1.44	I4	1.22	I4
3	1249	I4	2	I2	1.29	I3	390	I4	720	I3	1.06	I2	1.29	I4	1	I1
4	301	I1	1.11	I3	1.15	I3	160	I2	228	I1	1.06	I2	1.17	I4	1.13	I3
5	1230	I4	2.2	I2	1.22	I3	320	I4	301	I2	1.17	I4	1.33	I4	1.01	I1
6	277	I1	0.98	I4	0.3	I1	160	I2	230	I1	1.06	I2	1.23	I4	1.21	I4
7	503	I2	1.18	I3	0.4	I1	100	I1	201	I1	1.06	I2	1.16	I3	1.02	I2
8	701	I2	1.15	I3	0.38	I1	120	I2	150	I1	1.04	I2	1.08	I2	1.03	I2
9	258	I1	0.28	I4	0.27	I1	83	I1	272	I2	1.05	I2	1.01	I1	1.16	I3
10	920	I3	2.55	I1	2.1	I4	263	I3	241	I2	1.18	I4	1.44	I4	1.05	I2
11	930	I3	2.60	I1	1.98	I4	270	I3	826	I4	1.22	I4	1.5	I3	1.03	I1
12	260	I1	0.3	I4	0.2	I1	75	I1	273	I2	1	I1	1.13	I2	1.19	I4
13	338	I1	2.03	I3	1.97	I4	193	I3	320	I2	1.11	I3	1.38	I4	1.05	I2

**Figure 4:**
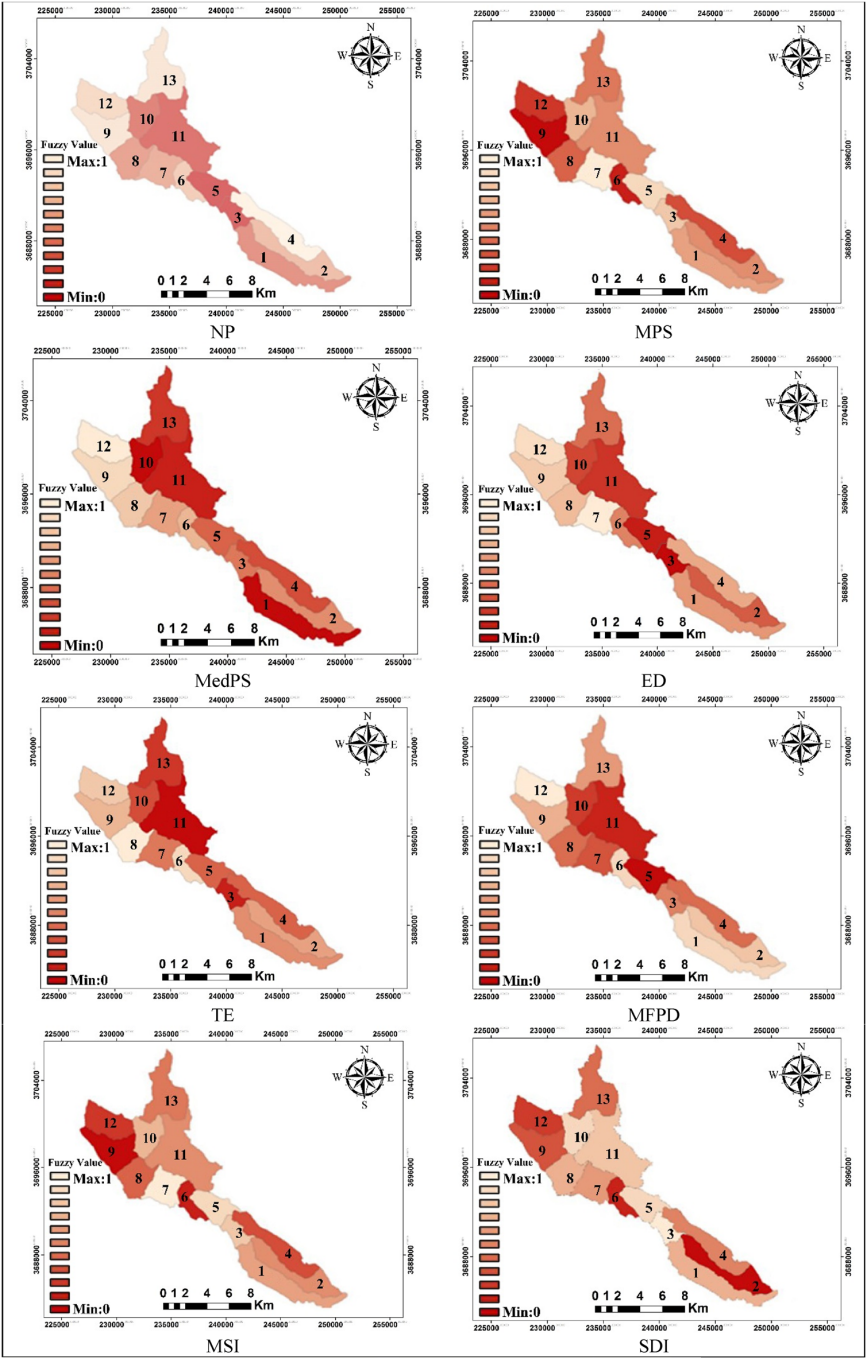
Standardized quantitative maps of sub-basins based on metrics.

The metrics for both the quantity and severity were computed for each sub-basin and presented in Table 2. Figure 4 displays the standardized quantitative metrics maps, which range from 0 to 1. The ecological degree metrics, activity intensity, and destruction values in Landscape were then determined for each sub-basin, based on the outcomes of Table 3. For sub-basin activity power computation, a value of 1 is assigned for the presence of human activity (I), while a value of 0 is used for the absence of development activity. The destruction coefficient for each sub-basin was computed to obtain the destruction values, and the median of the coefficients was utilized to classify the units.

**Table 3:** Determining the severity of activity, degree of vulnerability (V) and degree of destruction (LD) in landscape.

Sub-basin	(LD)	(V)	(ΣkI)
1	2.03	2	4.06
2	2.09	3	4.18
3	5.51	1	5.51
4	1.13	4	4.52
5	5.33	1	5.33
6	0.97	3	2.93
7	1.08	3	3.26
8	0.87	4	3.49
9	0.66	4	2.67
10	2.89	2	5.79
11	2.91	2	5.82
12	0.8	4	3.2
13	1.21	4	4.84

Table 3 indicates that the destruction intensity of the sub-basins is in the following order: 8 > 11 > 7 > 5 > 6 > 3 > 12 > 0 > 1 > 9 > 10 > 4 > 2. Table 4 presents values related to the destruction of Landscape, which determine the level of environmental degradation in the region. The destruction values were categorized based on standardization logic^23,24^. The results revealed that around 58.40% of the area is susceptible to development or further development, while only 8.38% of the area is suitable for conservation. However, approximately 33.24% of the area requires reconstruction, as per Table 4.

**Table 4:** Classification of study units based on landscape destruction coefficient.

Destruction class code	Fuzzy destruction range	Development and protection proportion	Area	
			ha	%
LD_1_	0–0.25	Prone to develop more	5676.61	37.96
LD_2_	0.25–0.5	Prone to develop	3056.39	20.43
LD_3_	0.5–0.75	Requires reconstruction	4973.06	33.24
LD_4_	0.75–1	Talented for protection	1254.25	8.38

**Figure 5:**
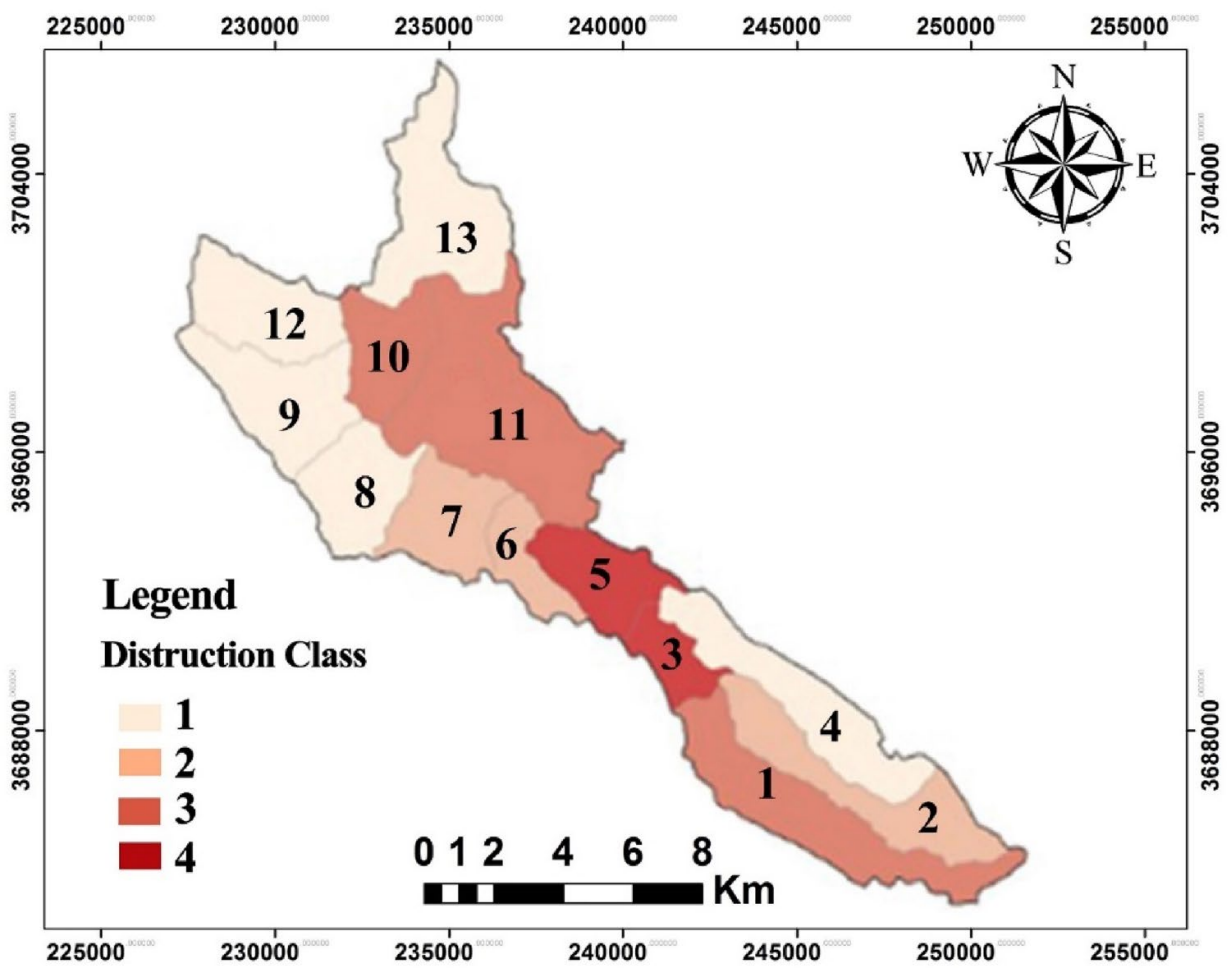
Map of the destruction sub-basins of Dadabad forest area.

The study’s findings indicate that in the sub-basins of Dadabad, the proportion of development and protection depends on the extent of landscape destruction (LD). Based on the results, sub-basins 3, 7, 8, 11, and 12 fall under class 1 in Fig. 5, which implies they have the highest proportion for development according to Table 5. Additionally, sub-basins 1, 5, and 6 are in class 2, which is appropriate for development, whereas sub-basins 0, 9, and 10 are in class 3 and require attention. Finally, sub-basins 2 and 4 are in class 4, which is of significant importance from a conservation standpoint, particularly ecological protection.

**Table 5:** Degrees of destruction in the landscape.

Number of sub-basin	1	2	3	4	5	6	7	8	9	10	11	12	13
Area (ha)	1642.59	1239.10	818.84	1336.4	435.41	916.92	916.92	502.25	1208.39	823.34	2507.13	1059.11	1573.44
Class code	L_D3_	L_D2_	L_D4_	L_D1_	L_D4_	L_D2_	L_D2_	L_D1_	L_D1_	L_D3_	L_D3_	L_D1_	L_D1_

## Discussion

### Comparison with previous studies

The findings of the study indicate that the destruction of forest areas is similar to previous studies conducted in Iran^25–27^, Poland^28^, Germany^29^, Brazil^30^, and Malaysia^31^. The main factor in this destruction is man-made activities such as road construction, which reduces natural areas, alters the function of the region, and decreases biodiversity^30,32^. Residential centers and population density are also significant factors in deforestation^33^. In this study, a novel hybrid version was used to model the destruction based on landscape ecology. The decision-making tool in the model is based on the metrics of intensity, vulnerability, and destruction values. The advantage of this model is that it determines the level of destruction in the landscape in a short time and with digital data. The results show that 40.88% of the total studied area is in a low degradation class and therefore more suitable for development. Additionally, 33.25% of the area needs to be rehabilitated, and 8.37% has been highly destructed and needs protection. The areas in the third class can be developed if there are no ecological constraints and will be protected if there is a restriction. Finally, sub-basins located farther away from residential centers, construction, and roads have experienced less damage^34,35^.

The findings of this study emphasize the urgent need for sustainable and responsible management of forest regions to protect the environment and preserve biodiversity. The significant impact of human activities on landscape destruction highlights the importance of considering ecological factors in development planning. The hybrid version of the destruction modeling used in this study provides a useful tool for decision-making in ecological restoration and protection projects. It enables the identification of priority areas for development, rehabilitation, and protection based on the level of destruction and vulnerability. The results suggest that areas farther away from residential centers, construction, and roads have experienced less damage, indicating the potential for sustainable development in these regions. This finding highlights the importance of considering the impact of human activities on the environment and the need for responsible development practices. The study’s findings can be useful for policymakers and land managers in developing strategies for sustainable and responsible land use practices, which would balance development with environmental preservation and protection.

### Research limitations

One limitation of our study is the reliance on available spatial datasets, such as satellite imagery and land cover maps, which have inherent limitations in resolution, accuracy, and temporal coverage. These limitations introduce uncertainty and may impact the precision of our analysis. Another limitation is the assumption of stationarity, assuming consistent relationships between landscape metrics and destruction over time. It is important to note that landscape dynamics can vary, and the observed relationships may not be applicable in different contexts or environmental conditions. Further research is necessary to explore the variability of these relationships in space and time.

### Policy recommendations

In terms of the future outlook for this study, there exist encouraging avenues for further research and development. Expanding the study to cover a broader geographic range or multiple forest ecosystems would enable the assessment of findings' generalizability. The inclusion of field data and validation would enhance the accuracy of the analysis. Furthermore, exploring advanced machine learning techniques and modeling approaches would contribute to improved predictive capabilities in assessing forest destruction. These directions offer valuable opportunities for further understanding and addressing the challenges associated with forest conservation and sustainable management.

## Conclusions

The study demonstrates the potential of landscape metrics in modeling forest area degradation, with sub-basins serving as natural units for assessing landscape destruction. The use of degradation coefficients to determine the development potential of sub-basins provides valuable insights for land use managers and planners in conserving natural landscapes, particularly forests. The findings of the study highlight the effectiveness of landscape metrics in assessing land destruction in natural areas and can contribute to informed decision-making processes for land use management and conservation. However, the study also identifies areas for improvement, such as the need to determine threshold limits for each land use, especially residential and agricultural use, in terms of area and to consider the socio-economic aspects of the model more carefully to make it more consistent with decision-making processes. Further research is necessary to determine the thresholds for each region, which requires separate studies, but the research experience has shown that it is possible. Overall, this study provides a valuable contribution to the field of land use management and conservation by demonstrating the potential of landscape metrics in assessing forest area degradation. It highlights the need for a more nuanced understanding of the factors influencing landscape degradation and the importance of considering socio-economic aspects in decision-making processes.

## Data Availability

The datasets generated and/or analysed during the current study are not publicly available due legal restrictions of Iran but are available from the corresponding author on reasonable request.
